# α‑Lipoic Acid and Deferoxamine Restore Redox–Metallomic Homeostasis in Dapsone Toxicity: Integrated Biochemical, Histological, and Molecular Modeling Evidence

**DOI:** 10.1021/acsomega.6c03045

**Published:** 2026-10-05

**Authors:** Rana Karen Mesquita da Silva, Amanda Caroline dos Santos Monteiro, Sara Kerolim Figueiredo Freitas, Ana Paula Holanda Santana, Kelly Davis, Wenddy Graziela Neves Lima, Pamela Suelen da Silva Seabra, Daniele Ferreira Sodré, Cristine Bastos do Amarante, Agnaldo da Silva Carneiro, Alessandro Quaresma Durães de Sousa, Savio Monteiro dos Santos, Antônio Rafael Quadros Gomes, Bruno Alexandre Quadros Gomes, Roseane Guimarães Ferreira, Marta Chagas Monteiro

**Affiliations:** † Laboratory of Oxidative Stress, Immunology and Microbiology, Faculty of Pharmacy, Health Science Institute, 37871 Federal University of Pará, Belém, Pará 66075110, Brazil; ‡ 521278 Laboratory of Chemical Analysis of the Museu Paraense Emílio Goeldi, Belém, Pará 66077-530, Brazil; § Computational Laboratory of Biological Systems, Faculty of Pharmacy, Health Science Institute, 37871 Federal University of Pará, Belém, Pará 66075110, Brazil

## Abstract

Dapsone (DDS), a sulfone antibiotic widely used in the treatment of leprosy and autoimmune diseases, is associated with hematotoxicity and hepatotoxicity due to its metabolic conversion into hydroxylamine derivatives that promote methemoglobinemia, iron dysregulation, oxidative stress, and tissue injury. In addition to CYP-mediated bioactivation, DDS may contribute to lysosomal stress-associated iron imbalance and oxidative propagation. Conventional antioxidants or iron-chelating therapies alone may provide only partial protection against these interconnected mechanisms. This study evaluated the protective effects of α-lipoic acid (ALA) and deferoxamine (DFO), administered individually or in combination, against DDS-induced oxidative and hematological disturbances using integrated *in vivo* and *in silico* approaches. Male Swiss mice received DDS (40 mg/kg, gavage) for 5 days, followed by treatment with ALA (25 mg/kg, gavage), DFO (40 mg/kg, i.p.), or their combination for an additional 5 days. Methemoglobin (MetHb), reduced glutathione (GSH), nitric oxide (NO), malondialdehyde (MDA), and plasma iron levels were evaluated. Histopathological analysis and molecular docking studies involving cytochrome P450 isoforms (CYP2C9, CYP2C19, CYP3A4, and CYP2E1) and hemoglobin were performed to investigate potential mechanisms underlying DDS-induced toxicity and the protective effects of ALA and DFO. DDS markedly increased MetHb, nitric oxide, malondialdehyde, and plasma iron levels, accompanied by depletion of GSH and hepatocellular degeneration. ALA and DFO individually attenuated oxidative and iron-related disturbances, whereas combined treatment effectively reduced methemoglobin formation and preserved hepatic architecture; however, plasma ferric iron levels remained comparable to those in DDS-treated animals. Molecular docking suggested preferential interactions of DDS with CYP2E1 and CYP3A4, supporting the biological plausibility of their involvement in oxidative bioactivation pathways, whereas DFO showed predicted interactions with CYP2C9, CYP2E1, and hemoglobin-associated iron sites. Collectively, these findings indicate that ALA and DFO each attenuate DDS-induced hepatotoxicity and hematotoxicity through distinct but complementary antioxidant, iron-chelating, and redox-modulating mechanisms. Combined treatment effectively improved selected endpoints, including methemoglobin formation and hepatic architecture, although its protective effects were endpoint-dependent. The molecular modeling results provide supportive mechanistic insights and should be interpreted as hypothesis-generating evidence complementary to the in vivo findings.

## Graphical abstract

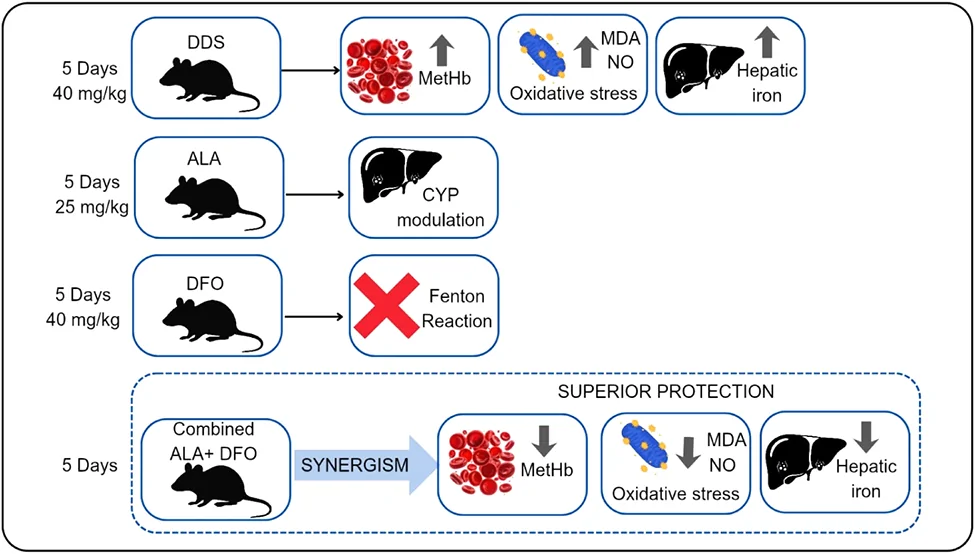

## 1 Introduction

Dapsone (4,4-diaminodiphenyl sulfone; DDS) is a synthetic sulfone widely prescribed for the treatment of leprosy and chronic inflammatory disorders due to its antimicrobial and anti-inflammatory properties. However, chronic DDS exposure is frequently associated with serious adverse effects, including methemoglobinemia, hemolytic anemia, sulfone hypersensitivity, hepatotoxicity, and neuroinflammation, which substantially limit long-term therapeutic adherence and patient safety.^1–3^ These toxic manifestations are primarily associated with the metabolic bioactivation of DDS by cytochrome P450 isoforms (CYP2C9, CYP2C19, CYP3A4, and CYP2E1), generating hydroxylamine derivatives such as dapsone hydroxylamine (DDS-NHOH) and monoacetyl dapsone hydroxylamine (MADDS-NHOH).^4^ These reactive metabolites act as potent oxidants capable of promoting hemoglobin oxidation (Fe^2+^ → Fe^3+^), redox-active iron accumulation, reactive oxygen species (ROS) generation, and lipid peroxidation, ultimately contributing to mitochondrial dysfunction and oxidative injury in multiple organs.^5,6^ Our research group has previously demonstrated, through both in vitro and in vivo approaches, that DDS metabolism induces pronounced oxidative imbalance and redox dysregulation. In patients undergoing multidrug therapy for leprosy, DDS exposure significantly increased methemoglobinemia (MetHb) and Heinz body formation while reducing catalase activity, indicating systemic oxidative stress accompanied by compensatory glutathione (GSH) upregulation.^7^ Experimental and molecular modeling studies have demonstrated the involvement of CYP2C19 in DDS oxidative bioactivation. Docking analyses revealed favorable interactions between DDS and the CYP2C19 catalytic cavity, whereas functional studies identified CYP2C19 as a major contributor to methemoglobin formation.^7,8^ In vitro exposure of human cells to DDS-NHOH promoted ROS generation, DNA fragmentation, and catalase inhibition, effects partially reversed by natural antioxidants such as resveratrol and α-lipoic acid (ALA).^9^ In vivo studies from our group also demonstrated that ALA effectively attenuated DDS-induced oxidative and hepatic injury in mice, reducing MetHb and ferric iron levels, restoring GSH and total antioxidant capacity (TEAC), and decreasing malondialdehyde (MDA) and liver enzyme alterations.^10^ Collectively, these findings established the antioxidant, anti-inflammatory, and mild metal-chelating properties of ALA as important contributors to the restoration of redox homeostasis during DDS-induced stress.

More recently, the neuroprotective dimension of ALA has also been elucidated. Chronic DDS administration induced iron accumulation and oxidative stress in the hippocampus and prefrontal cortex, accompanied by microglial activation, increased interleukin-1 beta (IL-1β), and reduced brain-derived neurotrophic factor (BDNF) expression 3. Similar neuroprotective effects of ALA against iron-associated oxidative stress and neuroinflammation have been reported in independent experimental models.^11^ Treatment with ALA reversed these alterations, restoring antioxidant defenses, increasing TEAC, attenuating astrocytic reactivity, and reducing neuroinflammatory markers. These observations indicate that ALA not only exerts systemic antioxidant effects but also modulates iron-associated neuroinflammatory pathways. Although oxidative bioactivation remains central to DDS toxicity, additional mechanisms associated with intracellular iron handling and organelle stress may also contribute to tissue injury. Beyond CYP-mediated metabolism, DDS may behave as a weak lysosomotropic base capable of accumulating within acidic endolysosomal compartments through ion trapping mechanisms.^12,13^ Weak-base lysosomotropic compounds may trigger lysosomal stress responses (LSR), characterized by lysosomal deacidification, release of redox-active iron, mitochondrial dysfunction, ROS amplification, and impairment of autophagic flux,^12,14,15^ Lysosomal destabilization may further amplify mitochondrial ROS production, establishing a feed-forward cycle between lysosomal dysfunction and oxidative mitochondrial injury. These interconnected events may contribute to iron dyshomeostasis and oxidative damage observed during DDS exposure, complementing the classical hydroxylamine-mediated toxicity pathway.^13,14^ In this context, ALA and deferoxamine (DFO) may exert complementary protective effects by preserving redox and lysosomal homeostasis. ALA contributes to mitochondrial and redox balance while supporting autophagic pathways through its antioxidant and redox-regulatory properties.^16,17^ In parallel, DFO may contribute to lysosomal stabilization by chelating redox-active iron pools and limiting Fenton-type oxidative propagation,^13,14,18^ Because DDS-induced toxicity involves persistent iron dysregulation and oxidative propagation that may not be fully controlled by antioxidant therapy alone, the integration of a specific iron chelator such as DFO may provide additional protection. Unlike ALA, which primarily regenerates endogenous antioxidant defenses and modulates redox-sensitive pathways, DFO limits catalytic iron availability for ROS propagation, stabilizes heme-associated iron, and may partially interfere with CYP-mediated oxidative metabolism^19,20^ Therefore, the present study investigated whether the combined administration of ALA and DFO could attenuate DDS-induced oxidative, hematological, and iron-related disturbances through complementary antioxidant and iron-chelating mechanisms. To address this hypothesis, we integrated in vivo biochemical and histopathological analyses with molecular docking simulations involving CYP isoforms and hemoglobin.

## 2 Results and Discussion

### 2.1 MetHb and Plasma Iron Levels

Administration of dapsone (DDS, 40 mg/kg) markedly increased the percentage of MetHb compared to the control group (11.2 ± 2.5% vs 1.6 ± 0.3%; *p* < 0.001) (Figure 1A). Treatment with ALA or DFO alone partially reduced MetHb formation (ALA: 3.1 ± 0.8%; DFO: 2.4 ± 0.9%, *p* < 0.001), whereas combined therapy (ALA + DFO) restored values close to those observed in the control group (1.7 ± 0.5%, *p* < 0.001).

**Figure 1:**
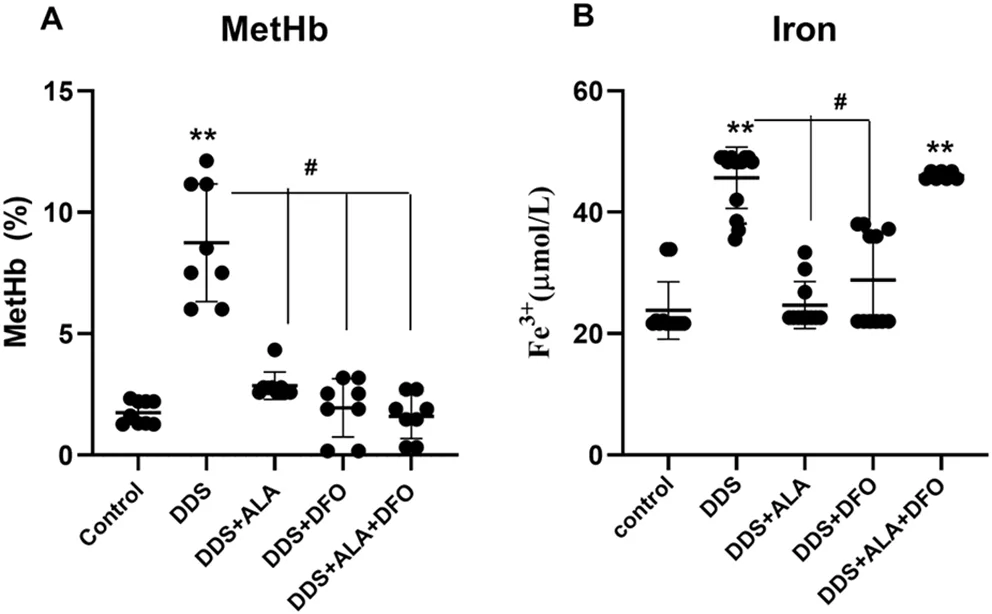
Effects of Dapsone (DDS) and the protective treatments α-lipoic acid (ALA) and deferoxamine (DFO) on (A) methemoglobin (MetHb %) and (B) plasma ferric iron (Fe^3+^, μmol/L) levels in mice. DDS markedly increased MetHb and Fe^3+^ concentrations compared with control. One-way ANOVA followed by Tukey’s post hoc test was used for comparisons: **p < 0.001 vs Control; ^#^ *p* < 0.001 vs DDS. Data are expressed as mean ± SD (*n* = 7). Abbreviations: ALA, α-lipoic acid; DFO, deferoxamine; DDS, dapsone; MetHb, methemoglobin; Fe^3+^, ferric iron.

Similarly, plasma Fe^3+^ concentrations significantly increased after DDS exposure (47.8 ± 6.9 μmol/L vs 21.5 ± 4.2 μmol/L in controls; *p* < 0.001) (Figure 1B). Treatment with ALA or DFO alone attenuated iron accumulation (ALA: 27.6 ± 5.4 μmol/L; DFO: 30.2 ± 6.1 μmol/L), whereas combined treatment with ALA + DFO did not reduce plasma ferric iron levels (45.3 ± 1.3 μmol/L), which remained comparable to those observed in the DDS group. These findings indicate that the iron-lowering effects observed with ALA and DFO monotherapies were not maintained when both agents were administered concurrently under the experimental conditions employed.

In addition to hydroxylamine-mediated oxidation, these effects may also involve lysosomal stress-associated iron release and mitochondrial ROS amplification triggered by the weak-base lysosomotropic behavior of DDS. The marked elevation of ferric iron and MetHb levels supports the participation of redox-active iron in DDS-induced oxidative injury, consistent with previous reports of hepatotoxicity and oxidative stress in patients chronically exposed to DDS.^12–14^

### 2.2 Hepatic GSH, NO, and MDA

Chronic exposure to dapsone (DDS, 40 mg/kg) induced marked oxidative imbalance in hepatic tissue, evidenced by depletion of GSH, elevation of nitric oxide (NO), and increased lipid peroxidation measured by MDA levels (Figure 2). Animals treated with DDS exhibited a significant reduction in hepatic GSH levels (91.8 ± 14.6 μmol/mL) compared to the control group (244.7 ± 52.7 μmol/mL; *p* < 0.05). Post-treatment with ALA or DFO alone substantially restored GSH concentrations (ALA: 201.1 ± 39.6 μmol/mL; DFO: 172.7 ± 44.9 μmol/mL, *p* < 0.001), indicating recovery of endogenous antioxidant defenses. In contrast, combined ALA + DFO treatment resulted in lower GSH levels (87.6 ± 33.9 μmol/mL), comparable to those observed in the DDS group. This finding suggests that the combined treatment did not enhance glutathione recovery under the experimental conditions employed. The distinct response observed with combined therapy indicates that the interaction between antioxidant and iron-chelating mechanisms did not enhance glutathione recovery under the experimental conditions employed.

**Figure 2:**
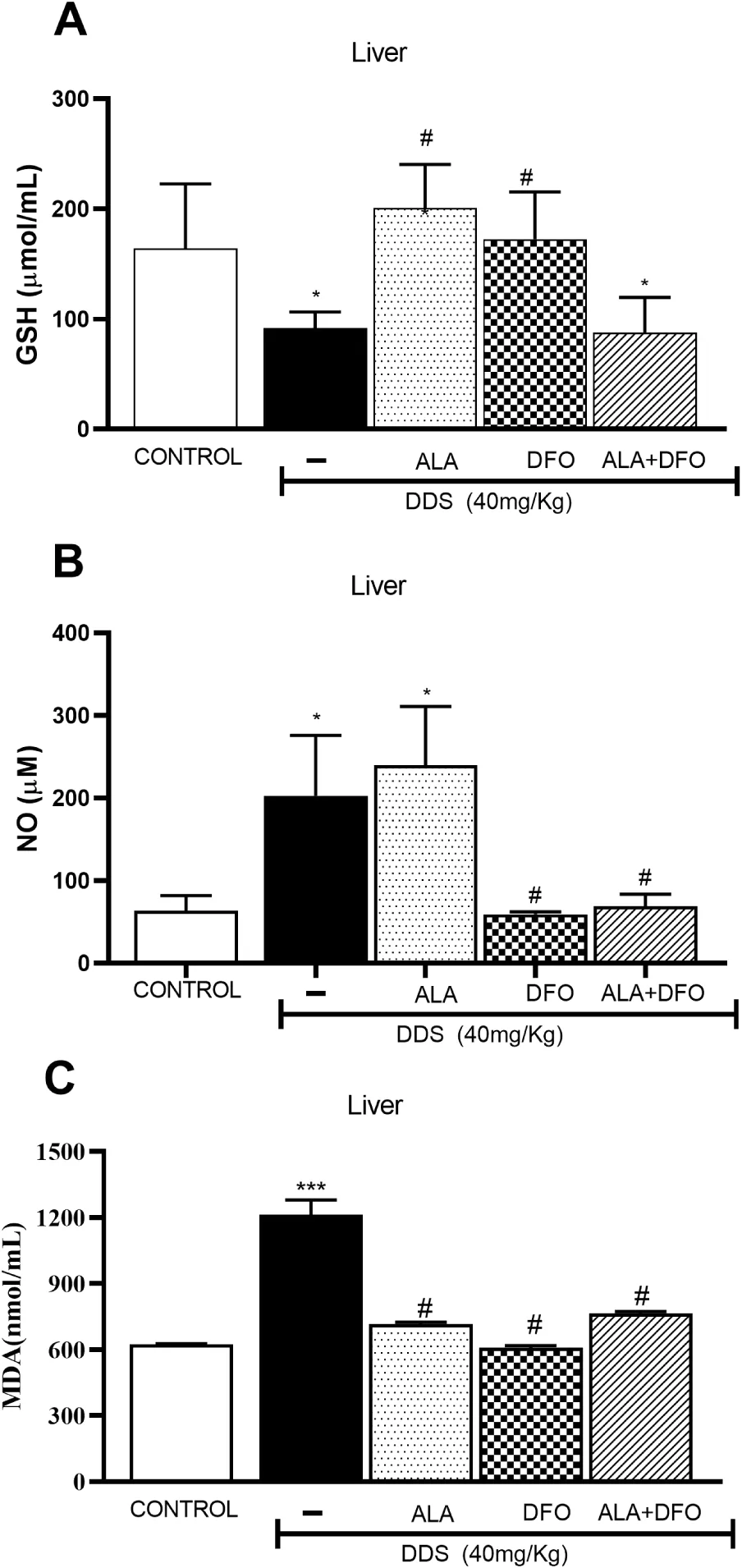
Effects of dapsone (DDS, 40 mg/kg) and post-treatment with α-lipoic acid (ALA, 25 mg/kg) and/or deferoxamine (DFO, 40 mg/kg) on hepatic oxidative biomarkers in mice. (A) Reduced glutathione (GSH); (B) nitric oxide (NO); and (C) malondialdehyde (MDA) levels. DDS induced a significant decrease in GSH and increases in NO and MDA relative to controls (*p* < 0.05). One-way ANOVA followed by Tukey’s post hoc test was used for comparisons; *p < 0.05 or ***p < 0.0001 vs Control; **^#^** *p* < 0.001 vs DDS. Data are expressed as mean ± SD (*n* = 7). Abbreviations: DDS, dapsone; ALA, α-lipoic acid; DFO, deferoxamine; GSH, reduced glutathione; NO, nitric oxide; MDA, malondialdehyde.

Hepatic NO levels significantly increased after DDS administration (179.2 ± 48.2 μM vs 103.8 ± 34.0 μM in controls; *p* < 0.05), confirming enhanced nitrosative stress. Elevated NO levels may reflect inducible nitric oxide synthase (iNOS) activation secondary to DDS-induced oxidative and inflammatory injury.^16,17^ ^,^ Excess NO can react with superoxide anion to generate peroxynitrite, thereby amplifying lipid peroxidation, mitochondrial dysfunction, and hepatocellular damage. Nitrosative stress may further interact with iron-dependent oxidative pathways, since peroxynitrite formation destabilizes mitochondrial membranes and amplifies ROS production. ALA treatment alone did not reduce hepatic NO levels (238.2 ± 56.0 μM), whereas both DFO and combined ALA + DFO therapy significantly reduced hepatic NO concentrations (DFO: 59.4 ± 2.5 μM; ALA + DFO: 69.0 ± 11.4 μM, *p* < 0.001), supporting an important contribution of catalytic iron to nitrosative propagation.

Similarly, MDA concentrations significantly increased following DDS treatment (1211.4 ± 67.6 nmol/mL) compared to controls (527.4 ± 4.4 nmol/mL; *p* < 0.0001). Administration of ALA or DFO alone markedly reduced MDA levels (ALA: 716.3 ± 8.4 nmol/mL; DFO: 608.2 ± 10.1 nmol/mL, *p* < 0.001), whereas combined treatment resulted in intermediate values (763.7 ± 9.9 nmol/mL). These findings indicate that both ALA and DFO attenuated DDS-induced lipid peroxidation, with DFO producing the greatest reduction in MDA levels. Although combined treatment also reduced lipid oxidative damage relative to DDS exposure, its effect was less pronounced than that observed with monotherapy under the experimental conditions employed.

### 2.3 Histopathological Evaluation of the Liver

Histological analysis of hepatic tissue revealed distinct morphological alterations among the experimental groups, demonstrating that chronic DDS administration induced marked structural liver injury, whereas post-treatment with ALA, DFO, or their combination preserved hepatic architecture to varying degrees (Figure 3A–E). The control group (Figure 3A) exhibited normal hepatic histomorphology at both 20× and 40× magnifications, characterized by preserved lobular organization, hepatocytes arranged in radial cords around the central vein, intact sinusoidal spaces, and the absence of inflammatory infiltrates or necrotic areas.

**Figure 3:**
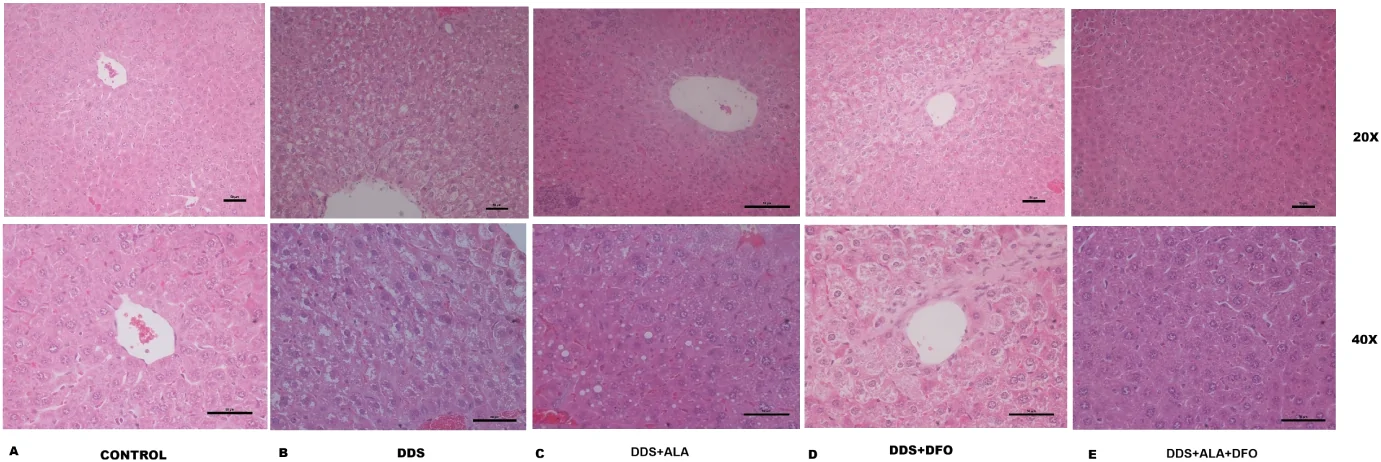
Representative photomicrographs of liver sections from mice treated with dapsone (DDS, 40 mg/kg) and post-treated with α-lipoic acid (ALA, 25 mg/kg), deferoxamine (DFO, 40 mg/kg), or their combination (ALA + DFO). Hematoxylin and eosin staining, 20× (top row) and 40× (bottom row). (A) Control: normal hepatic lobular structure with intact hepatocytes and sinusoids. (B) DDS: hepatocellular degeneration, vacuolization, focal necrosis, and sinusoidal congestion. (C) DDS + ALA: partial preservation of architecture and reduction in degenerative changes. (D) DDS + DFO: restored organization with fewer congested sinusoids and reduced degenerative alterations. (E) DDS + ALA + DFO: preserved hepatic architecture with reduced degenerative alterations. Scale bars = 50 μm (20×) and 25 μm (40×).

In contrast, the DDS-treated group (Figure 3B) displayed pronounced hepatocellular degeneration, including disorganization of hepatic cords, sinusoidal congestion, focal necrosis, cytoplasmic vacuolization, nuclear pyknosis, and karyolysis. Mild-to-moderate inflammatory infiltrates and occasional microvesicular steatosis were also observed, particularly in periportal and centrilobular regions. These histological alterations are consistent with oxidative and nitrosative tissue injury and closely parallel with the biochemical evidence of glutathione depletion and increased MDA and NO levels (Figure 2).

Animals treated with DDS + ALA (Figure 3C) exhibited partial preservation of hepatic structure, characterized by improved lobular organization, reduced sinusoidal congestion, fewer necrotic foci, and improved hepatocellular integrity, although mild vacuolization persisted in isolated cells. Likewise, the DDS + DFO group (Figure 3D) demonstrated substantial morphological recovery, including restoration of hepatic cord organization, reduced inflammatory infiltration, and decreased sinusoidal congestion. The combined-treatment group (DDS + ALA + DFO; Figure 3E) also exhibited preservation of hepatic architecture, with well-defined hepatic lobules, preserved hepatocellular morphology, minimal degenerative changes, and reduced inflammatory alterations. Collectively, these observations demonstrate that both monotherapy and combined treatment attenuated the structural hepatic damage induced by DDS exposure.

The histopathological findings paralleled the biochemical alterations observed in Figure 2, reinforcing the central role of oxidative stress and iron dysregulation in DDS-induced hepatotoxicity. The hepatoprotective activity of ALA is consistent with its well-established antioxidant properties, including intracellular redox cycling between α-lipoic acid and dihydrolipoic acid (DHLA), regeneration of endogenous antioxidant systems, modulation of mitochondrial function, and activation of Nrf2-dependent antioxidant pathways, which collectively contribute to the restoration of cellular redox homeostasis.^9,10,15–17^ Notably, our previous experimental study demonstrated that ALA significantly attenuated DDS-induced oxidative imbalance by restoring hepatic glutathione, reducing lipid peroxidation and methemoglobin formation, lowering ferric iron accumulation, and preserving liver architecture in vivo.^10^ The present findings corroborate and extend those observations by demonstrating that ALA also preserves hepatic histological integrity when evaluated alongside deferoxamine, while further highlighting the complementary but end point-dependent protective mechanisms of antioxidant and iron-chelating therapies. Furthermore, ALA has been reported to modulate intracellular iron metabolism by reducing the labile iron pool and regulating iron storage pathways, thereby contributing to attenuation of iron-dependent oxidative injury.^18^

Likewise, the hepatoprotective effects of DFO are consistent with its established ability to chelate redox-active iron, suppress ferroptosis, attenuate iron-dependent oxidative injury, and reduce inflammatory signaling in experimental models of liver disease.^19,23–25^ The marked reduction in hepatic NO and MDA levels observed in DFO-treated animals further supports the contribution of iron-dependent oxidative propagation to DDS-induced liver injury.

Although ALA and DFO exert complementary antioxidant and iron-chelating activities, their combined administration did not uniformly improve all biochemical end points. Whereas combination therapy effectively reduced methemoglobin formation, attenuated nitrosative stress, and preserved hepatic architecture, it failed to restore hepatic glutathione (GSH) concentrations and did not reduce plasma ferric iron levels to the extent observed with either monotherapy. These findings indicate that the interaction between ALA and DFO is more complex than a simple additive antioxidant response and appears to depend on the specific biological end point evaluated.

One plausible explanation for the observed GSH response is that ALA and DFO act through distinct but partially overlapping mechanisms. ALA directly promotes intracellular glutathione regeneration through its reduction to DHLA, recycling of endogenous antioxidants, and activation of redox-sensitive antioxidant pathways.^9,15–18^ In contrast, DFO primarily limits iron-catalyzed reactive oxygen species formation by reducing the availability of redox-active iron and suppressing Fenton-type reactions.^19,23–25^ Consequently, attenuation of oxidative stimuli by DFO may reduce the compensatory activation of glutathione synthesis normally stimulated by ALA, resulting in a less pronounced recovery of GSH despite overall attenuation of oxidative injury. Because this mechanism was not directly investigated in the present study, it should be regarded as a plausible mechanistic hypothesis requiring further experimental validation.

Although the present findings demonstrate protective effects of ALA and DFO under experimental conditions, their translation to clinical practice requires careful consideration. α-Lipoic acid has an established safety profile and has been widely used as an oral antioxidant at doses ranging from 300 to 600 mg/day in disorders associated with oxidative stress and peripheral neuropathy, where it is generally well tolerated in clinical studies.^9,10^ In contrast, deferoxamine is a clinically established parenteral iron chelator for the treatment of iron-overload disorders; however, prolonged administration requires careful monitoring because dose-dependent ocular toxicity, ototoxicity, growth impairment, and increased susceptibility to infections have been reported.^19,25,26^ Therefore, the doses employed in the present murine model should be interpreted as proof-of-concept experimental regimens rather than clinically equivalent therapeutic doses. Furthermore, the end point-dependent response observed with combined treatment indicates that concomitant administration does not necessarily confer additive protection across all biochemical parameters. Future pharmacokinetic, dose-optimization, and translational studies are warranted to establish the optimal therapeutic strategy for the individual or combined use of ALA and DFO in preventing or attenuating DDS-induced toxicity in clinical settings.^27^

### 2.4 Molecular Docking and Mechanistic Analysis

Molecular docking simulations were performed to investigate potential interactions between DDS, its hydroxylamine metabolite (DDS-NHOH), DFO, cytochrome P450 isoforms, and hemoglobin (Table 1). DDS exhibited the highest predicted binding affinity for CYP2E1 (−8.4 kcal/mol), followed by CYP3A4 (−7.2 kcal/mol). These computational findings are consistent with previous experimental studies demonstrating that DDS undergoes CYP-dependent oxidative bioactivation to hydroxylamine metabolites. Nevertheless, docking simulations estimate binding affinity rather than catalytic activity or metabolic conversion. Therefore, these observations should be interpreted as supportive computational evidence of the biological plausibility of CYP involvement rather than direct evidence that CYP2E1 or CYP3A4 catalyze DDS oxidation under the present experimental conditions.^1,2,8^ The predicted interactions are also compatible with the experimentally observed increases in methemoglobin and plasma ferric iron levels following DDS exposure (Figure 1). However, because molecular docking estimates ligand-binding affinity rather than enzymatic activity, catalytic turnover, or metabolic flux, these findings should be interpreted as computational evidence supporting the biological plausibility of CYP involvement rather than direct proof of enzyme participation. In particular, the proposed contribution of CYP2E1 remains a mechanistic hypothesis requiring confirmation through dedicated enzyme inhibition studies, recombinant CYP assays, and pharmacokinetic investigations.

**Table 1:** Molecular Docking Binding Affinities (Kcal/Mol) of Dapsone (DDS), Its Hydroxylamine Metabolite (DDS-NHOH), and Deferoxamine (DFO) with Cytochrome P450 Isoforms and Hemoglobin

Target protein	DDS	DDS-NHOH	DFO	Predicted pharmacological implication
CYP2C9	–6.6	–	–7.1	DFO showed higher predicted binding affinity than DDS, suggesting the possibility of interaction with regions involved in DDS binding. The functional significance of this prediction requires experimental validation.
CYP2C19	–6.3	–	–6.7	Comparable predicted affinities are consistent with a possible interaction of DFO with CYP2C19; however, the effect of this interaction on reactive metabolite formation remains unknown.
CYP3A4	–7.2	–	–6.4	DDS displays a stronger interaction, consistent with previous experimental evidence identifying CYP3A4 as an important enzyme involved in DDS oxidative metabolism.
CYP2E1	–8.4	–	–7.2	DDS exhibited the highest predicted affinity among the evaluated isoforms, consistent with previous reports implicating CYP2E1 in DDS bioactivation. However, this prediction should be interpreted as computational evidence requiring experimental validation.
Hemoglobin	–4.8	–5.6	–5.6	Both DDS-NHOH and DFO bind hemoglobin at comparable energies, suggesting that DFO may interact with heme-associated iron sites similarly to DDS-NHOH, which may contribute to limiting ferric iron accumulation and methemoglobin formation.

DFO demonstrated moderate to strong predicted binding affinities for CYP2C9 (−7.1 kcal/mol) and CYP2E1 (−7.2 kcal/mol), whereas its lower affinity for CYP3A4 (−6.4 kcal/mol) suggests limited interaction with this isoform under the docking conditions employed. These findings raise the possibility that DFO may influence DDS bioactivation pathways; however, the docking results alone cannot establish competitive inhibition or modulation of CYP catalytic activity and therefore should be interpreted cautiously pending experimental validation.

Importantly, DDS-NHOH and DFO exhibited comparable predicted binding energies toward hemoglobin (−5.6 kcal/mol), suggesting the possibility of interactions at heme-associated iron sites. These computational observations are consistent with the reduction in ferric iron accumulation and methemoglobin formation observed experimentally in DFO-treated animals (Figure 1). Likewise, the proposed attenuation of iron-dependent oxidative propagation is compatible with the recovery of hepatic redox biomarkers (Figure 2) and the preservation of liver architecture observed histologically (Figure 3). Nevertheless, these associations represent an integrated interpretation of the computational and experimental findings and should not be interpreted as direct mechanistic validation of the docking predictions.

Taken together, the docking analyses provide a computational framework that complements the biochemical and histopathological findings (Figures 1–3), supporting the biological plausibility of a multifactorial mechanism in which DFO may contribute to protection through iron chelation and potential modulation of oxidative pathways associated with DDS metabolism. These computational predictions should be interpreted in conjunction with the in vivo data and require further experimental validation before definitive mechanistic conclusions regarding CYP2E1 involvement or DFO-mediated modulation of DDS bioactivation can be established.

### 2.5 Visualization of Molecular Docking

Molecular docking simulations provided structural insights into the predicted interactions between DDS, its hydroxylamine metabolite (DDS-NHOH), DFO, cytochrome P450 isoforms, and hemoglobin (Figure 4A–E). In the CYP2C9 model (Figure 4A), DDS occupied the catalytic cavity through π-interactions and hydrogen bonding near the heme region, consistent with the proposed involvement of this isoform in DDS oxidative metabolism. DFO adopted a partially overlapping orientation, interacting predominantly with polar residues at the periphery of the active site, suggesting the possibility of interaction with regions involved in DDS binding. However, these structural observations do not establish competitive inhibition or modulation of CYP2C9 catalytic activity.

**Figure 4:**
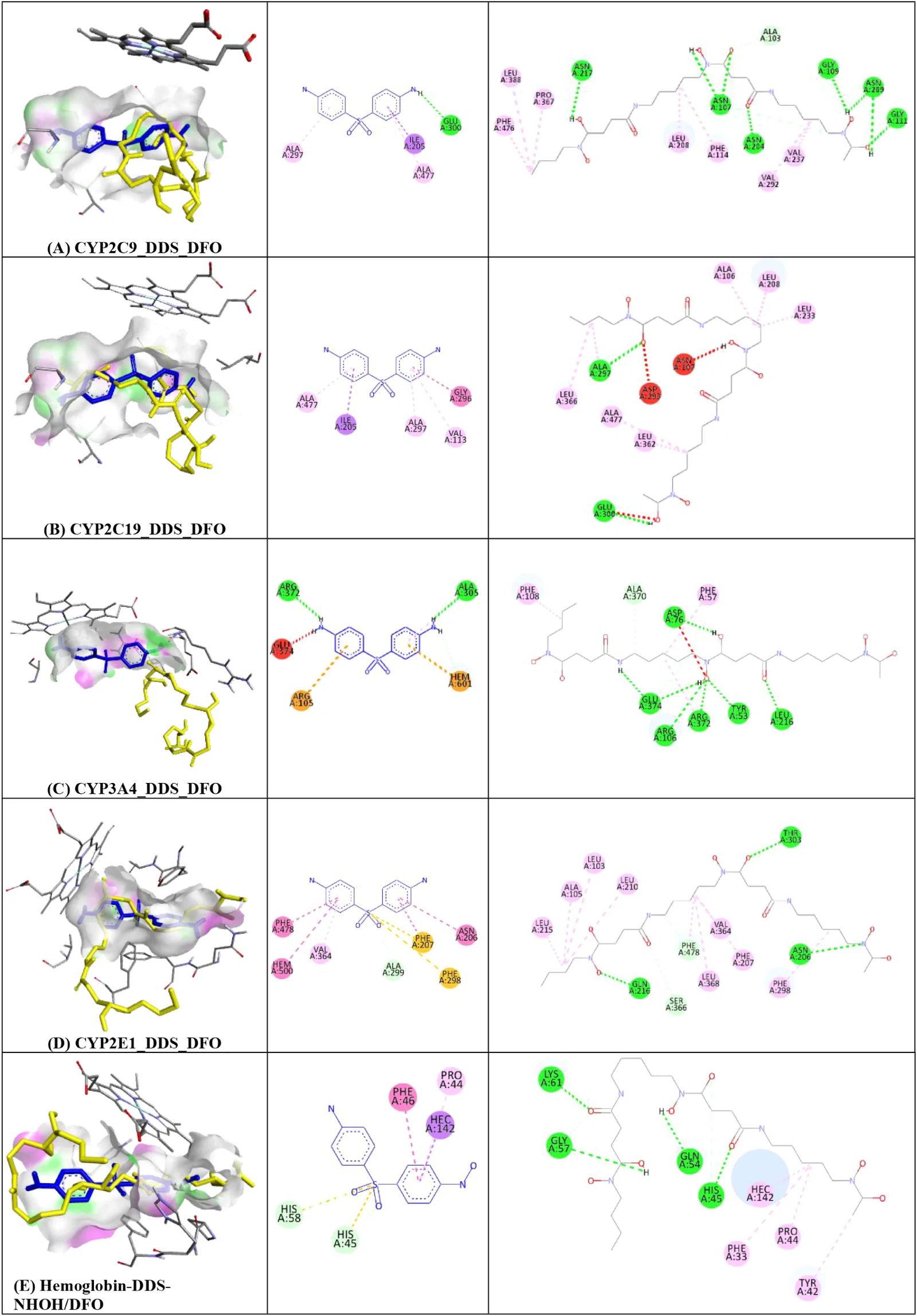
Molecular docking interactions of dapsone (DDS), its hydroxylamine metabolite (DDS-NHOH), and deferoxamine (DFO) with cytochrome P450 isoforms and hemoglobin. (A) CYP2C9–DDS/DFO complex; (B) CYP2C19–DDS/DFO complex; (C) CYP3A4–DDS/DFO complex; (D) CYP2E1–DDS/DFO complex; (E) Hemoglobin–DDS-NHOH/DFO complex. DDS shows high binding affinity with CYP2E1 and CYP3A4, consistent with its metabolic oxidation to DDS-NHOH. DFO displays predicted interactions with CYP2C9 and CYP2E1 that may be compatible with modulation of DDS bioactivation. In the hemoglobin model, DFO interacts with heme-associated iron sites and may interfere with DDS-NHOH interactions, potentially contributing to iron chelation and attenuation of methemoglobin formation. The docking visualizations provide computational evidence consistent with the proposed mechanism and should be interpreted as hypothesis-generating evidence requiring experimental validation. These computational findings complement the biochemical and histopathological results without constituting direct mechanistic proof. Abbreviations: DDS, dapsone; ALA, α-lipoic acid; DFO, deferoxamine; DDS-NHOH, dapsone hydroxylamine; Fe^3+^, ferric iron.

In the CYP2C19 complex (Figure 4B), both DDS and DFO exhibited moderate predicted binding stability. DDS interacted mainly through aromatic contacts, whereas DFO formed hydrogen bonds with polar residues surrounding the catalytic region. These predicted interactions are compatible with previous evidence indicating a contributory role of CYP2C19 in DDS metabolism and suggest the possibility of limited interaction by DFO under the docking conditions employed.

The CYP3A4 model (Figure 4C) demonstrated favorable accommodation of DDS within the enzyme cavity, involving stabilizing interactions with residues such as Ala305, Arg105, and Arg372, consistent with the established role of CYP3A4 in DDS oxidative biotransformation. In contrast, DFO exhibited weaker predicted binding and partial surface exposure, indicating limited interaction with this isoform. In the simulation, incomplete anchoring of DFO within the catalytic pocket was associated with higher predicted system energy, suggesting lower binding stability relative to DDS under the modeled conditions.

Among all evaluated isoforms, the CYP2E1 complex (Figure 4D) exhibited the highest predicted binding affinity for DDS (−8.4 kcal/mol), with anchoring near the heme iron through π-anion interactions. These computational findings are consistent with previous reports implicating CYP2E1 in DDS bioactivation; however, they should be interpreted as structural evidence supporting the biological plausibility of CYP2E1 involvement rather than direct demonstration of its catalytic role in DDS hydroxylamine formation. DFO interacted with adjacent polar residues, including Thr303 and Gln216, raising the possibility of interactions capable of influencing the local binding environment. Nevertheless, the functional significance of these predicted interactions remains to be established experimentally.

In the hemoglobin model (Figure 4E), DDS-NHOH and DFO exhibited comparable predicted interactions at heme-associated iron sites. DDS-NHOH interacted directly with the iron center, consistent with its established association with Fe^2+^ → Fe^3+^ oxidation and methemoglobin formation, whereas DFO established interactions with the heme region through hydroxamate-mediated coordination with ferric iron, consistent with its recognized iron-chelating properties. These structural observations are compatible with the reduction in ferric iron accumulation and methemoglobin formation observed experimentally (Figure 1), although the docking simulations alone cannot confirm the occurrence or extent of these molecular interactions in vivo.

Collectively, the docking visualizations provide a structural and computational framework that complements the biochemical and histopathological findings, supporting the biological plausibility of a multifactorial mechanism in which DFO may contribute to attenuation of DDS-induced oxidative injury through iron chelation and potential modulation of oxidative pathways associated with DDS metabolism. These computational predictions should be interpreted as hypothesis-generating evidence that complements, but does not replace, direct biochemical and enzymatic validation.

### 2.6 Interpretative Integration with Experimental Data

Molecular docking simulations complemented the biochemical and histological findings observed in vivo (Figures 1–3). DDS exhibited the highest predicted binding affinity for CYP2E1 (−8.4 kcal/mol) and CYP3A4 (−7.2 kcal/mol), consistent with previous reports implicating these isoforms in oxidative N-hydroxylation and DDS-NHOH formation.^1,2,4,8^ These interactions are consistent with the experimentally observed increases in MetHb formation, ferric iron accumulation, and oxidative stress biomarkers following DDS exposure^5,6^ However, these docking findings should be interpreted as computational evidence supporting the biological plausibility of CYP involvement rather than direct evidence of enzymatic participation.

DFO demonstrated moderate to strong affinity for CYP2C9 (−7.1 kcal/mol) and CYP2E1 (−7.2 kcal/mol), suggesting the possibility of partial interaction with catalytic regions involved in DDS metabolism. In parallel, the similar binding energies of DFO and DDS-NHOH toward hemoglobin (−5.6 kcal/mol) are consistent with potential interactions at heme-associated iron sites, which may contribute to the reduction of Fe^3+^ accumulation and methemoglobin formation observed experimentally.^29,30^

Integration of these in silico findings with the in vivo biochemical and histopathological data suggests that DFO may attenuate DDS-induced hepatotoxicity not only through iron chelation but also through potential modulation of oxidative DDS bioactivation pathways^22^–^25^. Concurrently, ALA contributes to restoration of intracellular redox balance, mitochondrial resilience, and antioxidant defenses,^14–18,20,21^ collectively reducing oxidative and nitrosative propagation.^21,31^

The combined experimental findings support a multifactorial model of DDS toxicity involving CYP-mediated reactive metabolite formation, iron dyshomeostasis, oxidative and nitrosative stress, and potentially lysosomal stress-associated mitochondrial dysfunction 12–14,32. Within this context, ALA and DFO exert complementary but end point-dependent protective effects through redox modulation, limitation of catalytic iron-dependent oxidative propagation, and preservation of hepatocellular integrity,^13,18,22^ Although the computational findings are consistent with the proposed mechanism, the contribution of CYP2E1 and the modulation of DDS bioactivation by DFO remain mechanistic hypotheses requiring further enzymatic and pharmacokinetic validation. Although lysosomal parameters such as lysosomal pH, autophagic flux, and lysosomal membrane integrity were not directly evaluated in the present study, the biochemical, histopathological, and computational findings are consistent with mechanisms associated with lysosomal stress responses induced by weak-base compounds,^12–14,32^

## 3 Materials and Methods

### 3.1 Ethical Statement and Experimental Design

All experimental procedures were conducted in accordance with the Guide for the Care and Use of Laboratory Animals established by the Brazilian National Council for the Control of Animal Experimentation (CONCEA) and the National Institutes of Health (NIH). The experimental protocol was approved by the Institutional Committee for Animal Ethics of the Federal University of Pará (protocol no. 6354310322).^28,29^

Adult male Swiss mice (*Mus musculus*), weighing 35–40 g and approximately 8 weeks old, were maintained under controlled environmental conditions (25 ± 1 °C; 12 h light/dark cycle) with free access to standard chow and water. Animals were randomly allocated into five experimental groups (n = 7 per group): (I) Control (10% sunflower oil); (II) DDS (40 mg/kg, gavage); (III) DDS + ALA (25 mg/kg, gavage); (IV) DDS + DFO (40 mg/kg, intraperitoneally); and (V) DDS + ALA + DFO.

DDS was administered for five consecutive days to induce oxidative and hematological toxicity, followed by post-treatment with ALA, DFO, or their combination for an additional 5 days. Twenty-four hours after the final treatment, animals were anesthetized with ketamine (150 mg/kg) and xylazine (10 mg/kg). Blood samples were collected by cardiac puncture, and liver tissues were excised for biochemical and histopathological analyses. Tissue samples were homogenized in phosphate-buffered saline (PBS; 1:10, w/v), sonicated, centrifuged at 3000 rpm for 10 min, and the resulting supernatants were stored at −80 °C until analysis.

### 3.2 Determination of MetHb

Methemoglobin levels were determined spectrophotometrically according to the method described by Evelyn and Malloy.^31^ Whole blood samples were diluted in phosphate buffer (pH 6.8), and absorbance was measured at 630 nm using a UV–Vis spectrophotometer. Total hemoglobin content was subsequently determined after potassium cyanide addition, and MetHb values were expressed as percentage of total hemoglobin.

### 3.3 Determination of Plasma Iron by Atomic Absorption Spectrometry

Plasma iron concentrations were determined using flame atomic absorption spectrometry (Thermo Scientific ICE 3500). Plasma samples were digested in a nitric–perchloric acid mixture under controlled heating conditions and appropriately diluted prior to analysis. Iron measurements were performed at 248.3 nm using ferric chloride (FeCl_3_) standard solutions for calibration. Plasma iron concentrations were calculated from the calibration curve and expressed as μmol/L.^32^

### 3.4 Oxidative Stress Analysis

Reduced glutathione (GSH) levels were determined using Ellman’s reagent [5,5′-dithiobis­(2-nitrobenzoic acid), DTNB] according to a previously described by Ellman.^33^ Samples were deproteinized with 2% trichloroacetic acid, centrifuged, and the resulting supernatants were reacted with DTNB. Absorbance was measured at 412 nm using a UV–Vis spectrophotometer, and GSH levels were expressed as μmol/mL.

Nitric oxide (NO) production was estimated indirectly by measuring total nitrite/nitrate concentrations using the Griess reaction.^34^ Equal volumes of sample and Griess reagent were incubated at room temperature for 10 min, and absorbance was measured at 540 nm. Nitrite concentrations were calculated from a sodium nitrite standard curve and expressed as μM.

Lipid peroxidation was assessed using the thiobarbituric acid reactive substances (TBARS) assay,^35^ and malondialdehyde (MDA) levels were used as an index of oxidative damage. Samples were incubated with thiobarbituric acid at 94 °C for 30 min, cooled to room temperature, and absorbance was measured at 535 nm. MDA levels were calculated from a standard calibration curve and expressed as nmol/mL.

### 3.5 Histopathological Analysis

Liver fragments were fixed in 10% buffered formalin, paraffin-embedded, sectioned at 5 μm thickness, and stained with hematoxylin and eosin (H&E). Histopathological evaluation was performed under light microscopy by qualitative assessment of hepatocellular degeneration, sinusoidal congestion, inflammatory infiltrates, vacuolization, and architectural disorganization at 20× and 40× magnifications.

### 3.6 Molecular Modeling and Molecular Docking Simulations

Molecular modeling and docking analyses were performed to investigate potential intermolecular interactions between dapsone (DDS), its hydroxylamine metabolite (DDS-NHOH), and deferoxamine (DFO) with biological targets associated with DDS bioactivation, oxidative stress, iron dysregulation, and methemoglobinemia. The selected receptor structures included cytochrome P450 isoforms involved in DDS metabolism (CYP2C9, CYP2C19, CYP3A4, and CYP2E1) and hemoglobin.

#### 3.6.1 Ligand and Receptor Preparation

Three-dimensional structures of DDS, DDS-NHOH, and DFO were constructed and geometrically optimized using Avogadro 1.2.0 and Gaussian 03 software.^36^ Energy minimization was performed using density functional theory (DFT) calculations employing the B3LYP hybrid functional and the 6–31G­(d, p) basis set to obtain energetically stable conformations.

Protein crystal structures were retrieved from the Protein Data Bank (PDB) under the following accession codes: CYP2C9 (4NZ2), CYP2C19 (4GQS), CYP3A4 (7LXL), CYP2E1 (3GPH), and hemoglobin (2H35). Crystallographic water molecules, cocrystallized artifacts, and nonessential ligands were removed using PyMOL v2.5. Polar hydrogens and Gasteiger charges were added using AutoDock Tools 1.5.6.

#### 3.6.2 Molecular Docking Simulations and Interaction Analysis

Docking simulations were performed using AutoDock Vina v1.2.3 to evaluate ligand–receptor interactions. Docking grids were centered on the catalytic heme regions of each receptor and extended to encompass the entire active-site cavity. For each ligand–receptor complex, 100 independent docking runs were performed using an exhaustiveness parameter of 8. The conformation exhibiting the lowest binding free energy (ΔG, kcal·mol^–1^) was selected for subsequent analyses.

Interaction analyses and structural visualizations were performed using Discovery Studio Visualizer 2.5 and LigPlot+ v2.2. Hydrogen bonds, hydrophobic contacts, π-interactions, and metal-coordination interactions involving heme-associated iron sites were evaluated.

#### 3.6.3 Validation of Docking Protocol

The docking protocol was validated by redocking the native cocrystallized ligands flurbiprofen (CYP2C9; PDB: 4NZ2) and ritonavir (CYP3A4; PDB: 7LXL) into their respective binding sites. Root-mean-square deviation (RMSD) values below 2.0 Å were considered indicative of satisfactory reproduction of the experimental binding mode and validation of the docking parameters employed. Validation was performed for CYP2C9 and CYP3A4, whereas docking results involving CYP2E1 and hemoglobin should be interpreted as supportive mechanistic evidence.

### 3.7 Statistical Analysis

Data were expressed as mean ± standard deviation (SD). Normality was assessed using the Shapiro–Wilk test. Statistical comparisons among experimental groups were performed using one-way analysis of variance (ANOVA), followed by Tukey’s multiple-comparisons post hoc test. Differences were considered statistically significant at *p* < 0.05. Statistical analyses were performed using GraphPad Prism version 10.0 (GraphPad Software, San Diego, CA, USA).

## Abbreviations

DDS: dapsone
DDS-NHOH: dapsone hydroxylamine
ROS: reactive oxygen species
MDA: malondialdehyde
DFO: deferoxamine
ALA: alpha lipoic acid
MetHb: methemoglobin
TEAC: trolox equivalent antioxidant capacity
SOD: superoxide dismutase
GSH: glutathione
NO: nitric oxide
DTNB: 5,5-dithiobis-2-nitrobenzoic acid
TBARS: thiobarbituric acid reactive substance
AST: aspartate aminotransferase
ALT: alanine aminotransferase
AP: alkaline phosphatase
H&E: hematoxylin and eosin
ANOVA: analysis of variance
DFT: density functional theory
CYP: cytochrome
CYP-2C9, CYP-2C19, CYP-3A4, CYP-2E1: cytochrome P450 isoforms
Fe^2+^: ferrous iron
Fe^3+^: ferric iron
BDNF: brain-derived neurotrophic factor
IL-1β: interleukin-1 beta
DHLA: dihydrolipoic acid
